# Investigation of transmigrated mandibular canines

**DOI:** 10.1590/2177-6709.24.6.065-068.oar

**Published:** 2019

**Authors:** Muhammad Azeem, Ambreen Afzal, Zubair Ahmed, Munawar Manzoor Ali, Arfan Ul Haq, Waheed Ul Hamid

**Affiliations:** 1 De’Montmorency College of Dentistry, Department of Orthodontics (Lahore, Pakistan).; 2 Altamash Institute of Dental Medicine, Department of Orthodontics (Karachi, Pakistan).; 3 Nishter Institute of Dentistry (Multan, Pakistan).; 4 University of Lahore, College of Medical and Dentistry (Lahore, Pakistan).

**Keywords:** Cuspid, Radiography, Panoramic, Orthodontics

## Abstract

**Introduction:**

Canine transmigration is a rare orthodontic condition and it is relevant to perform the proper diagnose at an early age.

**Objective:**

The aim of the current study was to find out the frequency of transmigrated mandibular canines (TRC) in orthodontic patients obtained from South Asian population of Pakistan origin.

**Methods:**

Panoramic radiographs of 2,550 untreated orthodontic patients (1,248 males; 1,302 females) were included, to investigate the presence, site, and type of TRC. Any permanent mandibular canine that was found to be crossing the midline in panoramic radiographs was considered as TRC.

**Results:**

The frequency of TRC was found to be 0.98%. TRC were only found unilaterally. No significance regarding gender and side was found. Nineteen TRC displayed a type 1 transmigratory pattern, while type 2 and type 5 transmigratory patterns were encountered in three patients.

**Conclusion:**

Frequency of transmigrated mandibular canines in the studied sample was 0.98%.

## RESUMO

**Introdução:**

a transmigração de caninos é uma condição ortodôntica rara, e é importante realizar seu adequado diagnóstico ainda em idade precoce.

**Objetivo:**

o objetivo do presente estudo foi avaliar a frequência da transmigração de caninos inferiores (TCI) em pacientes ortodônticos de origem paquistanesa.

**Métodos:**

com o objetivo de analisar a presença, o local e o tipo de TCI, foram avaliadas 2.550 radiografias panorâmicas pré-tratamento (1.248 homens, 1.302 mulheres) de pacientes ortodônticos. Foram considerados como TCI os caninos inferiores permanentes que, na radiografia, estivessem cruzando a linha média.

**Resultados:**

a frequência de TCI foi de 0,98%. Os achados demonstraram a presença de apenas TCI unilaterais, sem diferenças significativas em relação ao sexo e ao lado da arcada. O tipo 1 de transmigração foi encontrado em 19 pacientes e os tipos 2 e 5, em três pacientes.

**Conclusão:**

na presente amostra de origem paquistanesa, a frequência da transmigração de caninos inferiores foi de 0,98%.

## INTRODUCTION

The term “transmigration” refers to the intraosseous migration of unerupted teeth across the midline.^1^ ^,^ ^2^ Various authors defined transmigration in different ways. Javid^3^ and Mupparapu^4^ defined transmigration when more than 50% of total length of canine has crossed the midline, while Tarsitano et al.^18^ defined transmigration when the canine crosses the midline in its pre-eruptive phase.

The most common teeth affected by transmigration are mandibular canines. There are various theories proposed in literature regarding the etiology of transmigration, including the most accepted theory of abnormal displacement of tooth buds in embryogenesis phase.^5^ Other proposed aetiologies are inadequate space, early extraction or early loss of primary teeth, excessive crown length, genetics, endocrinal disorders, cystic growth, and other orofacial insults.^6^

The prevalence of transmigrated mandibular canines (TRC) is not constant and ranges from 0.33% to 0.46% in different population groups.^7^ ^,^ ^8^ To our knowledge, very few studies have been conducted so far regarding the calculation of frequency of TRC. The results may be different in different populations, because of genetic, racial or ethnic differences. Therefore, the aim of the current research was to calculate the frequency of transmigrated mandibular canines in orthodontic patients.

## MATERIAL AND METHODS

This study involved 2,550 panoramic radiographs from records of untreated orthodontic patients (1,248 males; 1,302 females) that visited public sector orthodontic centres during the years 2012-17. The sample was obtained from South Asian population of Pakistan origin. 

Inclusion criteria comprised: fully erupted permanent dentition except third molars, age range from 16 to 30 years, both genders, and acceptable quality of radiographs. Exclusion criteria were the following: subjects with cleft palate, orofacial syndromes or history of dentofacial trauma, and medically compromised subjects. 

The radiographs were examined by two blinded expert orthodontists at the same time, using standard light boxes. TRC was considered when the eruption path displaced to the other side of the arch, with at least 50% of total length of canine crown crossing the midline.^3^ ^,^ ^9^ TRC were classified according to Mupparapu’s classification (Table 1).^4^

**Table 1:** Mupparapu’s classification (2002).^4^

Type 1: Positioned mesioangularly across the midline within the jaw bone, labial, or lingual to the anterior teeth and with the crown portion of the tooth crossing the midline.
Type 2: Horizontally impacted near the inferior border of the mandible below the apices of the incisors.
Type 3: Erupting either mesial or distal to the opposite canine.
Type 4: Horizontally impacted near the inferior border of the mandible, below the apices of either the premolars or molars on the opposite side.
Type 5: Positioned vertically in the midline (the long axis of the tooth crossing the midline) irrespective of eruption status.

**Figure 1:**
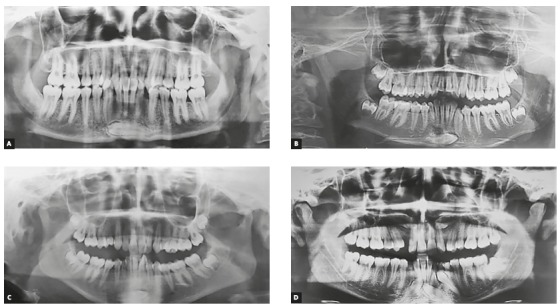
Patterns of transmigrated mandibular canines.

The frequency of transmigrated mandibular canines (Fig 1) was calculated and presented as percentages. Subject’s sex, age, side of involvement and number of transmigrated mandibular canines were recorded. Fisher’s exact test was used to test the effect of gender on TRC. 

Two evaluators were calibrated until the achievement of intraevaluator reliability and reproducibility. Kappa statistic was applied for analyzing the intra evaluator agreement. Interevaluator disagreement was corrected by consensus and agreement. To assess reliability, 24 days after the initial assessment, 300 radiographs were randomly selected and reassessed by the same two evaluators. A paired sample t-test was applied and results between the first and second evaluation were found to be reliable. 

## RESULTS

The mean age of the sample was 23.10 ± 4.10 years. A total of 25 (0.98%) out of 2,550 (100%) displayed TRC: 9 (36%) were males (23.41 ± 4.86 years), 16 (64%) were females (23.36 ± 4.71 years), with no significant gender differences (*p*= 0.919) (Table 2). 

**Table 2:** Descriptive statistics of gender, side and pattern in TRC patients (n = 25).

	n (Percentage)	P-value
Male	9 (36%)	0.919
Female	16 (64%)	
Left	15 (60%)	0.834
Right	10 (40%)	
Unilateral	25 (100%)	0.001*
Bilateral	0 (0%)	
Type 1	19 (76%)	0.003*
Type 2	3 (12%)	
Type 5	3 (12%)	

*Statistically significant.

All transmigrated canines presented unilaterally (*p*= 0.001) with no significant differences between the right *versus* left side (*p*= 0.834) (Table 2). In addition, 60% (n= 15) TRC were found on the left side, while 40% (n= 10) TRC were found on the right side. Nineteen TRC displayed type 1 transmigratory pattern (*p*= 0.003) (Table 2). 

## DISCUSSION

The frequency of transmigrated mandibular canine is not constant and is found to be distinct in different populations. Prevalence rates of TRC have been found to be 0.33% to 0.46% in various population groups.^7^ ^,^ ^8^ Therefore, the aim of the current research was to calculate the frequency of transmigrated mandibular canines in orthodontic subjects. The present study showed frequency of TRC to be 0.98%, with no significant gender differences, and all the TRC were found to be unilateral (Fig 1). 

The results of the present study are not comparable with other population studies.^10^ ^,^ ^11^ Aktan et al^10^ in a study on Turkish population found 0,34% of TRC incidence. Javid et al^3^ found only one TRC in his research on one thousand students, while Zvolanek et al.^12^ did not find any TRC in a sample of 4000 subjects. In a study by Sharma and Nagpal,^13^ the incidence of TRC was found to be 0.66% in a sample of 3000 Indian subjects. In a study by Kamiloglu and Kelahmet,^14^ in Cypriote population, a prevalence of 0.44% TRC was found. The frequency of transmigrated canines in the present study was found to be higher, i.e., 0.98%, which can be linked to the genetic, racial and ethnic differences.

Mupparapu^4^ introduced the classification of transmigrated canines and divided TRC into five types, according to the angulation and positioning of TRC relative to midline on panoramic radiographs. In the present study, the same classification system was followed to find out the pattern of distribution of TRC. The results showed that type 1 transmigratory pattern was the most prevalent. Our results are in accordance with the study by Sharma^13^ who also found the type 1 to be the most common type of TRC in Indian population.

In present study no significant differences were found regarding the distribution of TRC in both the genders, and in right versus left side. This is in contrast with findings of previously conducted studies where TRC was found to be more prevalent in females and on left side,^10^ ^,^ ^11^ and in contrast with the findings of Aydin^7^ and Sharma and Nagpal,^13^ in which TRC was found to be more common in males. The transmigrations are more common in the mandibular arch and this can be linked to voluminous symphysis, increased labial inclination of mandibular incisors, and increased conical root-crown morphology of lower cuspids.^13^

Literature is unclear regarding the etiology and mechanism of occurrence of TRC.^15^ ^-^ ^17^ The most accepted theory so far, regarding etiology and mechanism of occurrence of TRC, is atypical drifting of the lamina tissue during the embryogenesis.^5^ There are several reported factors associated with the occurrence of TRC, such as: early primary teeth loss, ectopia of permanent cuspids, over-retained primary canines, lack of space, local dental trauma to follicular tissues of canines, and cystic changes.^6^

## CONCLUSION

» Frequency of transmigrated mandibular canines was 0.98%.
» Type 1 transmigratory pattern was most prevalent.
